# Fragmentation of the Habitat of Wild Ungulates by Anthropogenic Barriers in Mongolia

**DOI:** 10.1371/journal.pone.0056995

**Published:** 2013-02-20

**Authors:** Takehiko Y. Ito, Badamjav Lhagvasuren, Atsushi Tsunekawa, Masato Shinoda, Seiki Takatsuki, Bayarbaatar Buuveibaatar, Buyanaa Chimeddorj

**Affiliations:** 1 Arid Land Research Center, Tottori University, Tottori, Japan; 2 Institute of Biology, Mongolian Academy of Sciences, Ulaanbaatar, Mongolia; 3 World Wide Fund for Nature (WWF) Mongolia, Ulaanbaatar, Mongolia; 4 School of Veterinary Medicine, Azabu University, Sagamihara, Japan; 5 Mongolia Program, Wildlife Conservation Society (WCS), Ulaanbaatar, Mongolia; Université de Sherbrooke, Canada

## Abstract

Habitat loss and habitat fragmentation caused by anthropogenic activities are the main factors that constrain long-distance movement of ungulates. Mongolian gazelles (*Procapra gutturosa*) and Asiatic wild asses (*Equus hemionus*) in Mongolia are facing habitat fragmentation and loss. To better understand how their movements respond to potential anthropogenic and natural barriers, we tracked 24 Mongolian gazelles and 12 wild asses near the Ulaanbaatar–Beijing Railroad and the fenced international border between Mongolia and China between 2002 and 2012. None of the tracked gazelles crossed the railroad, even though gazelles were captured on both sides of the tracks at the start of the study. Similarly, we did not observe cross-border movements between Mongolia and China for either species, even though some animals used areas adjacent to the border. The both species used close areas to the anthropogenic barriers more frequently during winter than summer. These results suggest strong impacts by the artificial barriers. The construction of new railroads and roads to permit mining and other resource development therefore creates the threat of further habitat fragmentation, because the planned routes will divide the remaining non-fragmented habitats of the ungulates into smaller pieces. To conserve long-distance movement of the ungulates in this area, it will be necessary to remove or mitigate the barrier effects of the existing and planned roads and railroads and to adopt a landscape-level approach to allow access by ungulates to wide ranges throughout their distribution.

## Introduction

Long-distance movements of large herds of ungulates are one of the most spectacular ecological phenomena, yet these movements are endangered [1], [2]. Habitat loss and habitat fragmentation by anthropogenic activities are the main factors leading to the disappearance of long-distance movements by ungulates. Such activities have potentially severe consequences, and may cause regional extinctions or a drastic population decline of some ungulates [3], [4].

Central Asian grassland is one of the largest remaining ecosystems where ungulates move over long distances. These animals include the saiga antelope (*Saiga tatarica*), the Asiatic wild ass (*Equus hemionus*), and the Mongolian gazelle (*Procapra gutturosa*), which are some of the last abundant wild ungulates in the world [5], [6]. Mongolian gazelles inhabit the grasslands of Mongolia, northern China, and southern Russia [7], [8]. The current population is estimated to be between 0.4 and 2.7 million [9], and a mega-herd of more than 200 000 gazelles was observed in 2007 [10]. Annual movement distances of Mongolian gazelles exceed 1000 km in some cases [11], [12], [13], and changing vegetation conditions are considered to be the main factor that governs their seasonal movements and the resulting distribution [10], [11], [12], [14], [15]. Mongolian grasslands and semi-desert ecosystems are also the last intact habitat of Asiatic wild asses and are home to nearly 80% of the global population (ca. 43 000 in 1997) [16]. The movement patterns of Mongolian gazelles [12], [13], [17], and of Asiatic wild asses [18] are considered more nomadic than the typical seasonal migration between specific winter and summer ranges. Thus, a conservation strategy that will give these animals access to a wide range of their habitat is needed.

Mongolian gazelles and Asiatic wild asses are currently facing potentially severe habitat fragmentation and loss. Tracking data for ungulates in Mongolia have suggested that habitat fragmentation is being caused by the Ulaanbaatar–Beijing Railroad for Mongolian gazelles [19] and for Asiatic wild asses [20] and by the international border between Mongolia and China for Mongolian gazelles [12], [17] and for Asiatic wild asses [18], [20], although one wild ass was reported to have crossed the border fences [18]. Furthermore, new mining projects and railroad and road construction are creating additional barriers in the habitat of these ungulates. Therefore, it is important to evaluate the strength and influence of barrier effects to support efforts to conserve long-distance movement of ungulates.

However, previous studies of the barrier effects had small sample sizes and short tracking periods, especially near the Ulaanbaatar–Beijing Railroad: only two gazelles for 1 year [19] and one wild ass for less than 1 year [20]. In addition, all of these animals were captured and tracked only on the southeastern side of the railroad. A carcass survey along the railroad reported that gazelle carcasses were found both outside and inside the fences that surround the tracks, suggesting that the fences were not a complete barrier [21]. Based on these limited previous results, it is clearly desirable to better understand the strength of the barrier effect. To provide this understanding, the following data are necessary: the percentage and frequency of crossing the railroad for large groups of animals tracked for long periods, and the possible locations where they crossed the barriers. Unfortunately, obtaining such data is logistically challenging in the vast area because tracking many animals takes a huge expense.

To evaluate the barrier effects of the Ulaanbaatar–Beijing Railroad and fences along the international border, we captured Mongolian gazelles in areas on both sides of the railroad, near the tracks, and tracked them for 3 years. We also analyzed the movements of other gazelles and wild asses that were captured in areas more distant from the Ulaanbaatar–Beijing Railroad to evaluate other potential barriers, such as another railroad located in northeastern Mongolia, the international border, rivers, and mountains.

## Materials and Methods

### Ethics statement

All datasets were collected within the framework of the legal requirements of Mongolia and Japan. Capture and collaring of Mongolian gazelles and Asiatic wild asses was conducted under an agreement between Tottori University, Japan, and the Institute of Biology, Mongolian Academy of Sciences, that was signed on 29 June 2007. Standard practices for the protection of animal research subjects were followed by the guidelines of the American Society of Mammalogists for the use of wild mammals in research [22].

### Study area

The study area was a steppe and desert steppe area in southern and eastern Mongolia. The region is characterized by a high elevation (a mean of about 1000 m above sea level). The climate is strongly continental and arid and is characterized by cold winters (with minimum temperatures below −35°C), dry and windy springs, and relatively wet and hot summers (with maximum temperatures above 40°C). Annual precipitation increases from about 100 mm in the southern desert steppe to 300 mm in the northern steppe [23]. Fine-leafed grasses and *Allium* spp. dominate the steppe vegetation, and semi-shrubs, shrubs, and some grasses dominate the desert steppe regions.

The main distribution area of the Mongolian gazelle is southeastern half of Mongolia. The Ulaanbaatar–Beijing Railroad bisects the main distribution area of the Mongolian gazelle. The nearly linear railroad runs northwest to southeast through Mongolia, and barbed-wire fences have been built alongside it to prevent accidents involving livestock and wild animals [19], [21]. The distribution area of Asiatic wild asses in Mongolia located drier and more western region than that of Mongolian gazelles. Distribution of the two species partially overlapped in western side of the Ulaanbaatar–Beijing Railroad, and the railroad cuts off the wild ass habitat, where wild asses have basically disappeared in the east side (P. Kaczensky, unpublished data).

Barbed-wire fences have also been built along international borders to China and Russia. The typical fences of railroads and borders are ca. 1.2 m high with 3-horizontal-line barbed wires. Mongolian gazelles are able to pass under such fences, and some gazelle crossings were observed [24]. However, there are regional variations of height and number of horizontal lines and vertical lines are added in some areas.

### Gazelle and wild ass tracking

From 2002 to 2007, we captured and collared 33 Mongolian gazelles and 16 Asiatic wild asses within an area of ca. 423 000 km^2^ in southeastern Mongolia. The 1-year tracking data for the gazelles captured in 2002 were previously reported [11], [19]. Some of this data suggested the presence of a barrier effect caused by the Ulaanbaatar–Beijing Railroad. To evaluate this effect in more detail, we captured eight gazelles on the southeastern side and eight on the northwestern side of the railroad in 2007. The gazelles were captured using at least two cars and motorbikes, and a 200–300-m-long net that was 1.5 m tall. The vehicles chased the herd of gazelles slowly toward the net, where they were captured. We used anesthesia to capture the wild asses [25].

Each animal was collared with a satellite transmitter (model ST-18, ST-20 Telonics Inc., Mesa, AZ, USA). The collar weighed 550 to 663 g (A-3210) for the gazelles and 1005 g (A-3310) for the wild asses. Details of the collar performance were reported in [26]. The transmitters were programmed to transmit radio signals at intervals of 7 to 8 days. The location data were recorded by the French Collecte Localisation Satellites service (CLS, http://www.cls.fr/).

Accuracy of the location data was given as location classes (LCs) ranging from 3 to 0. Their estimated positional errors were <250 m for LC 3, 250 to 500 m for LC 2, 500 to 1500 m for LC 1, and >1500 m for LC 0 [27]. To evaluate the barrier effects of the railroad and the international border, we selected only data from LCs 3, 2, and 1 to avoid using data with questionable accuracy. Table 1 summarizes the tracking period, capture sites, and number of days with positioning in LC 1 or better for each animal. In our analysis, we only used the data for gazelles that were tracked for more than 100 days. For Asiatic wild asses, we used all individuals with LC≥1 for at least 1 day, because all transmitters for the wild asses stopped sending data within 401 days as a result of unknown problems, probably broken antennas [24], and this limited the amount of data available for the analysis.

**Table 1:** Information on the capture and tracking of the ungulates in the present study.

Species	ID	Sex	Age	Date of capture	Site of capture		Region	Estimated date of death	Last date of location data	Tracking duration (total days)	Days with LC≥1
					Lat. (°N)	Long. (°E)					
Mongolian gazelle	37571	F	-	2002/10/18	45.553	109.474	SW	-	2006/1/18	1188	129
	37572	F	-	2002/10/18	45.553	109.474	SW	-	2005/5/22	947	128
	37573	F	-	2002/10/26	43.955	103.179	west	2003/11/11	-	381	50
	37574	F	-	2002/10/29	43.981	103.030	west	2003/3/3	-	125	36
	41243	F	-	2003/7/24	45.055	108.540	SW	-	2006/5/23	1034	121
	25363	F	-	2003/8/13	46.889	114.699	east	-	2007/7/18	1435	146
	42645	F	-	2003/8/18	48.277	113.684	east	-	2006/7/29	1076	136
	25381	M	-	2003/11/12	47.118	114.900	east	2005/2/12	-	458	64
	25448	M	3?	2004/7/28	47.680	115.486	east	-	2005/12/24	514	45
	67920	F	1	2007/5/18	46.518	109.036	NE	2008/4/28	-	346	20
	67921	F	2	2007/5/18	46.518	109.036	NE	2010/2/13	-	1002	115
	67923	M	1	2007/5/22	45.661	110.895	NE	-	2008/12/8	566	33
	67931	F	1	2007/5/22	45.661	110.895	NE	-	2009/6/18	758	63
	67932	F	Ad	2007/5/22	45.661	110.895	NE	-	2008/7/17	422	35
	67925	F	1	2007/5/29	45.210	109.071	SW	-	2008/7/25	423	29
	67926	M	1	2007/5/29	45.210	109.071	SW	-	2008/6/15	383	24
	67933	F	Ad	2007/5/29	45.210	109.071	SW	2008/7/25	-	423	40
	67927	M	2	2007/5/31	46.648	108.084	SW	-	2009/4/7	677	79
	67928	F	Ad	2007/5/31	46.648	108.084	SW	-	2010/3/1	1005	98
	67929	M	1–2	2007/6/1	46.648	108.084	SW	-	2008/10/29	516	56
	78510	F	2.5	2007/11/1	43.420	109.115	south	-	2012/3/30	1611	113
	78511	F	2.5	2007/11/1	43.420	109.115	south	-	2012/3/30	1611	162
	78512	F	4–5	2007/11/1	43.420	109.115	south	2008/2/16	-	107	4
	78513	M	1(0.5)	2007/11/2	43.420	109.115	south	2009/3/6	-	490	38
	67936	M	3	2007/6/20	43.310	109.218	south	-	2007/10/27	129	15
	67937	M	8–9	2007/6/20	43.410	109.205	south	-	2007/12/30	193	21
	67940	F	4–5	2007/6/20	43.401	109.212	south	-	2008/7/18	394	7
	67941	M	8	2007/6/21	43.413	109.243	south	-	2007/6/29	8	1
	67943	F	3	2007/6/20	43.392	109.208	south	-	2008/2/8	233	4
	67945	F	9–10	2007/6/20	43.419	109.147	south	-	2008/2/24	249	9
	67946	M	5	2007/6/21	43.398	109.200	south	-	2007/9/9	80	3
	67947	F	9–10	2007/6/21	43.389	109.183	south	-	2008/6/28	373	11
	67948	M	2–3	2007/6/21	43.389	109.184	south	-	2007/10/3	104	2
	67949	F	9–10	2007/6/20	43.423	109.177	south	-	2008/7/25	401	5
	67950	F	4	2007/6/20	43.420	109.216	south	-	2007/9/1	73	4
	67951	M	9–10	2007/6/21	43.382	109.226	south	-	2007/12/14	176	4

**Figure 1:**
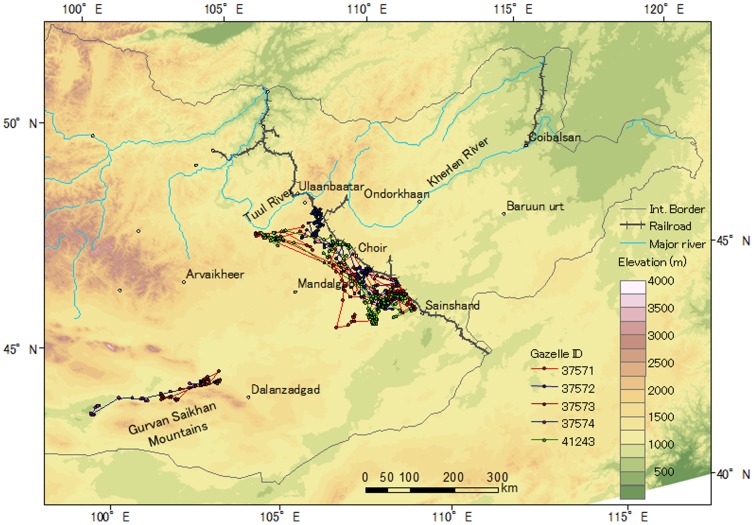
Movements of the tracked Mongolian gazelles captured on the southwestern side of the Ulaanbaatar–Beijing Railroad in 2002 and 2003. Table 1 provides the basic information and tracking period for each gazelle.

**Figure 2:**
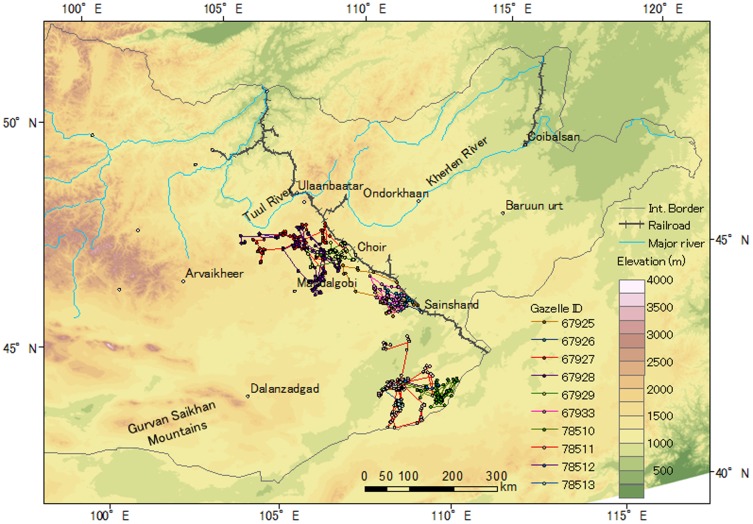
Movements of the tracked Mongolian gazelles captured on the southwestern side of the Ulaanbaatar–Beijing Railroad in 2007. Table 1 provides the basic information and tracking period for each gazelle.

**Figure 3:**
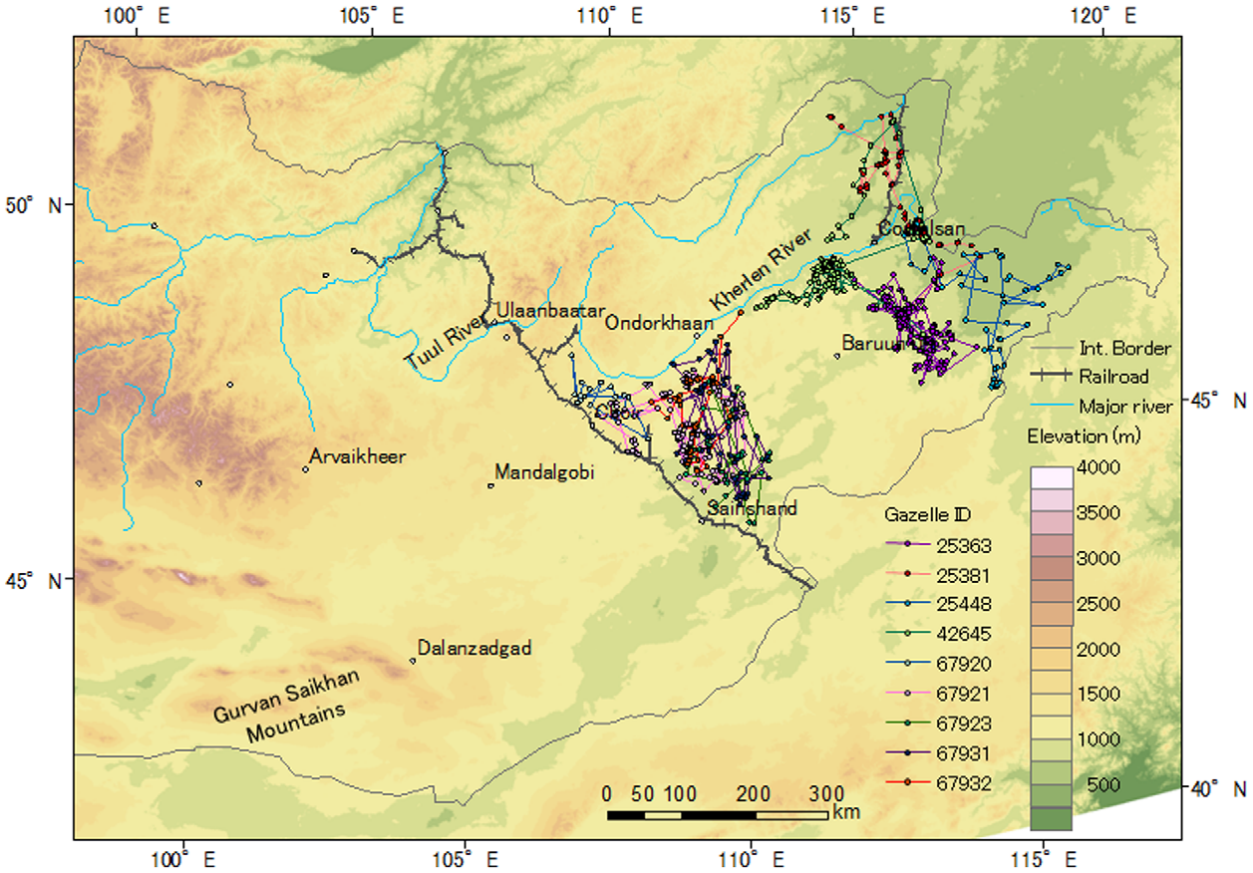
Movements of the tracked Mongolian gazelles captured on the northeastern side of the Ulaanbaatar–Beijing Railroad in 2007. Table 1 provides the basic information and tracking period for each gazelle.

**Figure 4:**
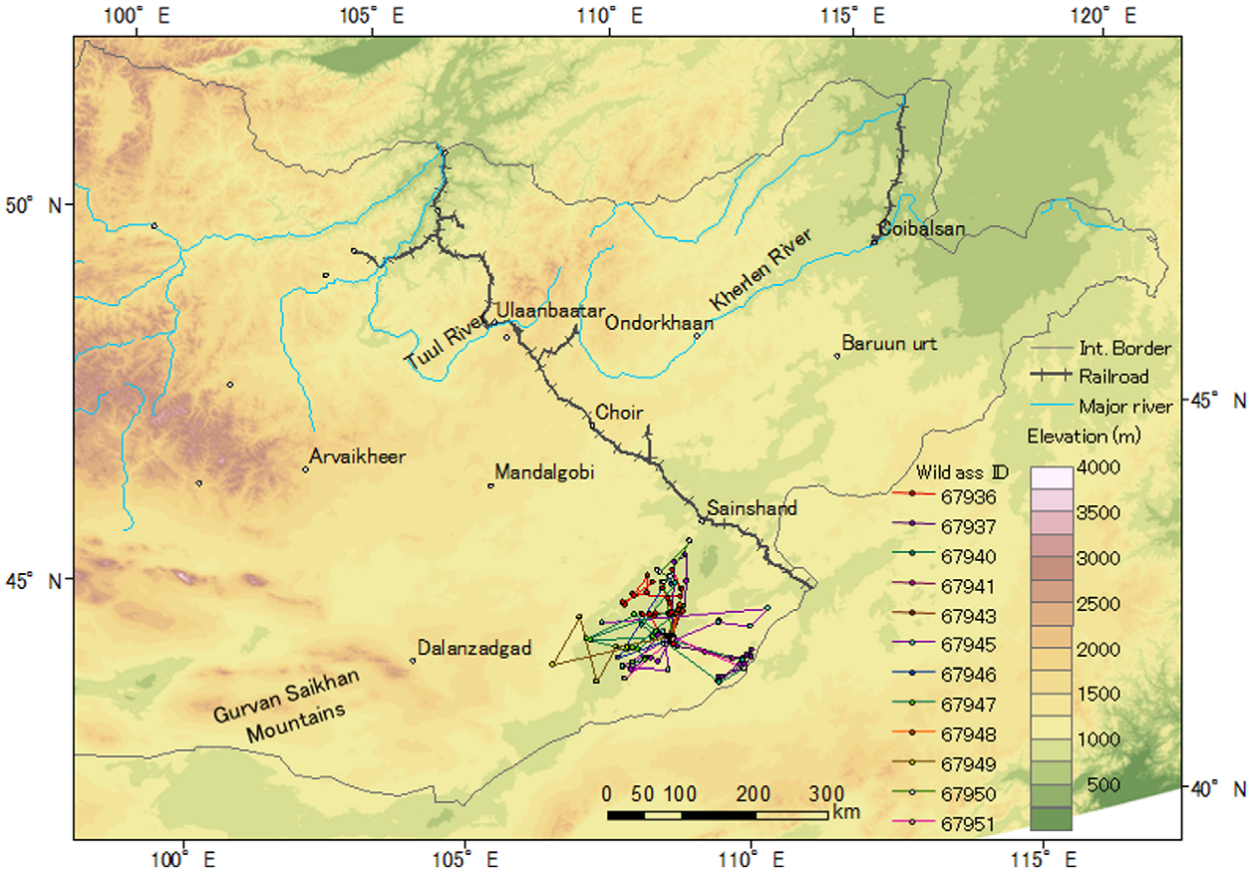
Movements of the tracked Asiatic wild asses in Mongolia. Table 1 provides the basic information and tracking period for each wild ass.

The regions SW and NE indicate that gazelles were captured southwest or northeast, respectively, of the Ulaanbaatar–Beijing Railroad (Figs. 1, 2, 3, 4). Other directions indicate the relative area within the animal's overall distribution. LC≥1: location class 1 (a positioning error of 500 to 1500 m) or more accurate location classes.

We analyzed data from gazelles that were tracked for more than 100 days (*n* = 24) and all wild asses that sent usable location data (*n* = 12), and selected the best ranking data from each day according to the LC to plot the animal movements. When two or more positions were recorded within the same best ranking LC on a given day, we selected the last location in that class to avoid effects of autocorrelation between locations of the same individual. We overlapped the movement data on a digital map of the study area, and calculated distances between each location of animals and the anthropogenic barriers using the ArcGIS software (Environmental Systems Research Institute Inc., Redlands, CA, USA). For gazelles that located within 10 km from the railroads or borders at least 1 day, we tested monthly differences of ratio located within 10 km from the anthropogenic barrier to all location data by one-way repeated measured ANOVA. For wild asses, statistical analysis was not conducted because of limited sample size.

## Results

We tracked 8 of the gazelles for more than 3 years (Table 1). Eight gazelles were estimated to have died during the tracking, and the tracking of the other 16 gazelles ended when the transmitter battery ran out of power (Table 1). Tracking of the Asiatic wild asses was interrupted early, so we only obtained a small amount of accurate location data from each wild ass, although this data suggested that all of the wild asses continued to move until the last location data were sent (Table 1).

Sixteen gazelles used close area within 10 km to the Ulaanbaatar–Beijing Railroad or borders, but no gazelles crossed these barriers (Figs. 1, 2, 3). Three Asiatic wild asses moved close to the border between Mongolia and China, but they also did not cross the border (Fig. 4).

Two gazelles (IDs 25381, 42645) crossed another railroad with a lower traffic level and incomplete fencing in the northeastern area and the 35–75-m wide Kherlen River, where reaches depth of up to 3.5 m (G. Davaa, pers. comm.) (Fig. 3). One gazelle (ID 25381) crossed the river in mid-June, and the other (ID 42645) crossed the river between late November and early December, when the river was frozen. No gazelle crossed the 90–250-m wide Tuul River, where reaches depth of up to 3.8 m (G. Davaa, pers. comm.).Two gazelles (IDs 37573, 37574) captured in the southwestern region moved along the base of the Gurvan Saikhan Mountains, but did not climb to higher elevations (Fig. 1).

**Figure 5:**
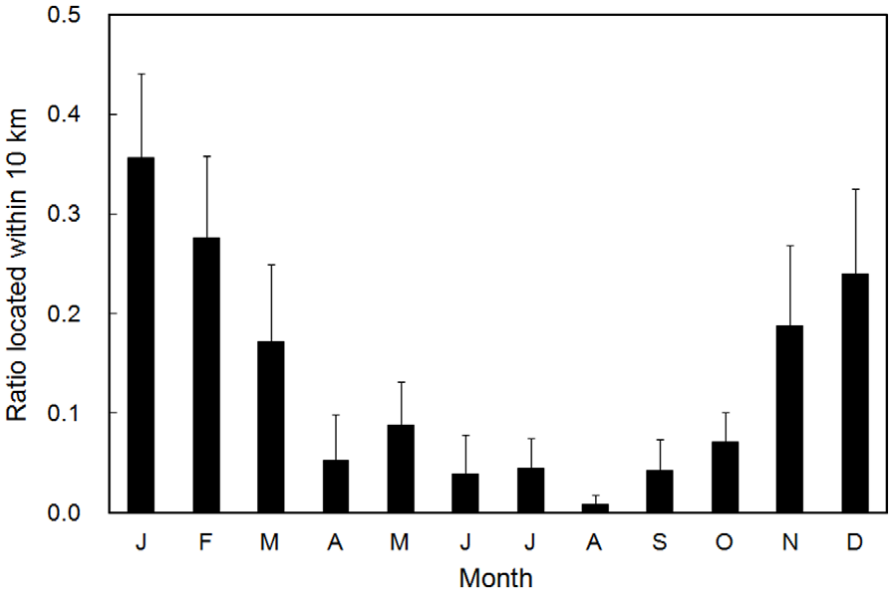
Monthly ratio (mean+SE) located within 10 km from the anthropogenic barriers for the tracked Mongolian gazelles that used areas within 10 km from the barriers at least once (*n* = 16).

**Figure 6:**
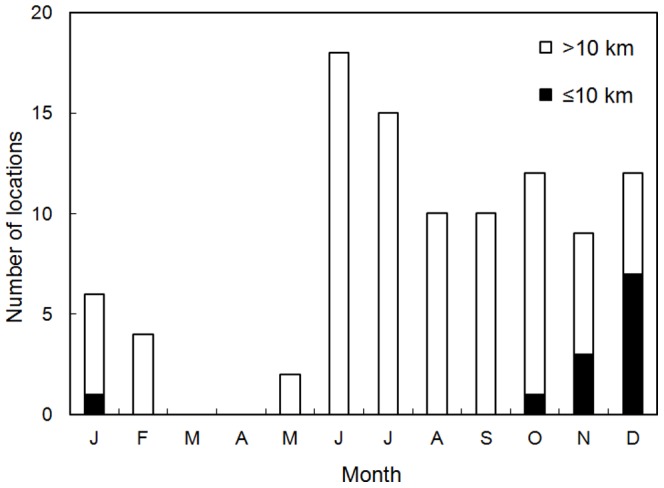
Monthly numbers of locations within 10 km and over 10 km from the anthropogenic barriers for the tracked Asiatic wild asses.

For the gazelles used the areas within 10 km from the anthropogenic barriers, ratio located within 10 km were different seasonally (*p*<0.001), that is, higher during winter than summer (Fig. 5). The wild asses used areas within 10 km from the barriers only from October to January (Fig. 6).

## Discussion

### Barrier effects

The results suggest a strong barrier effect caused by the Ulaanbaatar–Beijing Railroad and the international border. None of the gazelles that we tracked for several years crossed the railroad, even though some gazelle crossings were reported [24] and some gazelle carcasses were found trapped in the fences [21]. A barrier effect created by the fenced international border was also revealed, as has been reported previously in other studies of Mongolian gazelles [13], [17] and Asiatic wild asses [20], although one wild ass crossed the border fences between Mongolia to China and back [18]. Some of the gazelles and wild asses tracked in the present study reached the border, but none crossed it. These barrier effects would be similar for another gazelle species, the goitered gazelle (*Gazella subgutturosa*), that inhabits our study area and travels long distances, although there are no usable movement data for this species.

Two gazelles used areas close to another railroad located in northeastern Mongolia and moved along the railroad during some periods, and both gazelles also crossed the tracks and moved far beyond them. This railroad is not completely fenced on both sides. In addition, the frequency of trains along the tracks is less than that along the Ulaanbaatar–Beijing Railroad. These factors would let the gazelles easily cross the tracks.

Two large rivers, the Kherlen River and the Tuul River, and mountains may also have created a barrier effect, although two gazelles crossed the Kherlen River, even when the river is not frozen. No gazelle crossed the Tuul River, but this river is close to the current northern limit of the Mongolian gazelle's distribution in the region [9]. Both the river itself and other environmental factors, such as topography, climate, and high human density, would affect gazelle movements and the species' distribution in the region. In addition to natural movement barriers that have existed for evolutionally time scale and would have affected ungulates distributions, such as mountains and rivers, creating new anthropogenic barriers must fragment ungulates habitats.

The gazelles and wild asses used close areas to the anthropogenic barriers more frequently during winter than summer. Winter is the food shortage period in a year for the ungulates. Therefore, ungulates would seek areas where plants are more available during winter, and be impeded to move ahead by and wonder along the barriers. It may cause higher mortality of ungulates due to that ungulates cannot reach areas of enough plant availability to survive until spring. In this case, the barrier effect is serious not only for conservation of ungulates' movements but also for conservation of ungulates' population. Higher plant availability inside the fences may be another possible explanation for the use near barriers by the ungulates during winter because plants inside fences had not grazed by livestock and wild animals until winter. Even in the case, however, merits for ungulates may be not so much because such area is quite limited comparing to ungulates' distribution area and there is risk injuring ungulates by barbed wires. In fact, many carcasses were found along and inside fences [21]

### Conservation implications

Anthropogenic structures appear to have been fragmenting the habitats of ungulates in Mongolia and interfering with their long-distance movement. Planned railroads and roads to permit mining and other forms of resource development may therefore create a severe threat of further habitat fragmentation if similar fences to the existing railroads and borders are created, because the planned routes will further divide the remaining intact habitats of the Mongolian gazelle, Asiatic wild ass, and goitered gazelle. In addition to physical barriers, a high human density has a negative impact on habitat selection by the Mongolian gazelle [28]. Therefore, mining activities would make wild ungulates avoid the areas close to mines and worker settlements and may further restrict free access of ungulates to wide areas of their range. Mining and other resource extraction activities would therefore create additional potential threats of habitat fragmentation, and may further constrain or even eliminate long-distance movements of these animals, potentially leading to regional extinction or a drastic population decline, as has been reported for many other ungulates [3].

Under the climate conditions in Mongolia, ungulates need easy access to wide ranges. Interannual variability of climate and vegetation conditions is large, because all of their habitats in our study area are located in arid land [29], [30], and extreme conditions such as drought and severe winters can lead to high aggregation of Mongolian gazelles in a small area with superior forage resources [10]. Similar problems have led to mass mortality of re-introduced Przewalski's horse (*Equus ferus przewalskii*) in Mongolia [18]. The movement patterns of Mongolian gazelles [12], [13], [17] and Asiatic wild asses [18], [31] in Mongolia are considered to be nomadic rather than the more typical seasonal migration pattern between summer and winter ranges, suggesting that the ungulates adapt to their highly fluctuating and unpredictable environment by moving when necessary [12], [13], [32], although the data to support this hypothesis are still quite limited. Habitat fragmentation and habitat loss are serious threats to the long-distance movement of Mongolian ungulates, and this may lead to high mortality rates when adverse environmental conditions in one area force the animals to search for more suitable rangeland. A landscape-level approach will therefore be required to give these animals access to the wide ranges they require for survival and to conserve the animals.

No regional genetic isolation has been reported for the Mongolian gazelle [33], [34], [35], but two subpopulations were identified by genetically for wild asses in Mongolia, for which is likely explained by mountain ranges [20]. Therefore, if anthropogenic barriers prevent ungulates' movements for long time, it may cause new genetic subpopulations of ungulates. Genetic disturbance by anthropogenic barriers is another serious concern for wildlife.

For the Ulaanbaatar–Beijing Railroad, fences on both sides of the railroad are a major cause of the barrier effect [19], [21]. To let ungulates cross the tracks, removal of the fences would be effective. Even though it would be impractical to remove all of the fences, establishing areas without fences at some appropriate interval would be effective, because some of the tracked gazelles moved long distances along the railroad, and other gazelles crossed another railroad with fewer fences in the northeastern part of the study area. Modifying the fences (e.g., raising the lowest wire) might be effective, because the gazelles can cross these barriers, but larger livestock, such as horses, cows, and camels, cannot [24]. Creating new passages for the animals, such as the over- and under-passes created along the Qinghai–Tibetan Railway in China [36] and along highways in North America [37], [38], [39], would be also effective, but this approach would be expensive. For new railroads that are currently being planned or are under construction, no fence zones or passages for animals with a suitable structure should be created at frequent intervals. Solving the problem created by the barrier effect at an international border such as the one between China and Mongolia would be a more complicated and challenging issue. International conservation strategies such as the creation of transboundary protected areas are desirable because they would help to conserve the long-distance movement required by ungulates in this region's highly fluctuating and unpredictable environment.
